# Utilising artificial intelligence in prehospital emergency care systems in low- and middle-income countries: a scoping review

**DOI:** 10.3389/fpubh.2025.1604231

**Published:** 2025-06-20

**Authors:** Odhran Mallon, Freddy Lippert, Willem Stassen, Marcus Eng Hock Ong, Caitlin Dolkart, Thomas Krafft, Eva Pilot

**Affiliations:** ^1^Faculty of Health, Medicine and Life Sciences, Maastricht University, Maastricht, Netherlands; ^2^Falck, Copenhagen, Denmark; ^3^Faculty of Medical Sciences, Newcastle University, Newcastle upon Tyne, United Kingdom; ^4^Faculty of Health and Medical Sciences, University of Copenhagen, Copenhagen, Denmark; ^5^Division of Emergency Medicine, University of Cape Town, Cape Town, South Africa; ^6^Department of Emergency Medicine, Singapore General Hospital, Singapore, Singapore; ^7^Rescue.co, Nairobi, Kenya; ^8^Flare Emergency Services, Nairobi, Kenya

**Keywords:** artificial intelligence, machine learning, prehospital, emergency medical services, LMIC, emergency patient care

## Abstract

**Introduction:**

Improvements in prehospital emergency care have the potential to transform patient outcomes globally, but particularly within low-and middle-income countries. Whilst artificial intelligence is being implemented in many healthcare settings, little is known about its use in prehospital emergency care systems. This scoping review aims to uncover how artificial intelligence is currently being used within the prehospital emergency medical services of low-and middle-income countries and assess the implications for future development.

**Methods:**

A review of peer-reviewed articles using any artificial intelligence models in prehospital emergency care in low-and middle-income countries was carried out. Medline, Global Health, Embase, CINAHL and Web of Science were searched for studies published between January 2014 and July 2024. Data were extracted, collated and presented in table format and as a narrative synthesis. This scoping review is reported using the PRISMA-ScR guidelines.

**Results:**

Sixteen articles were included in the study. Most studies were conducted in China and deep learning models were used in half of the studies. Articles assessing dispatch forecasting were the most common, although artificial intelligence tools are also utilised in classification and disease prediction. There was significant variation in sample sizes throughout the selected studies. Overall, machine learning algorithms outperformed other comparator methods when they were used in all but two studies.

**Discussion:**

Limitations included only analysing articles published in English. Additionally, studies that did not identify the model as an artificial intelligence tool, or did not explicitly mention a LMIC in the title or abstract may have been inadvertently excluded. Whilst artificial intelligence can significantly benefit patient care in out-of-hospital settings, the continued development of this technology requires proper consideration for the local sociocultural contexts and challenges in these countries, along with using complete, population-specific datasets. Further research is needed to support advancements in this field and promote the realisation of universal health coverage.

**Systematic review registration:**

https://doi.org/10.17605/OSF.IO/9VS2M, osf.io/9vs2m.

## 1 Introduction

Despite its recent explosion into the public imagination, artificial intelligence (AI) has actually played a defined role in healthcare for over half a century (1). Research into the use of AI within healthcare systems is accelerating at an ever-increasing rate. As a result, AI continues to shape medical practise across more disciplines than ever before (1). In the context of global health, researchers believe that AI may play a vital role in realising many of the United Nations’ Sustainable Development Goals (SDGs) (2), particularly targets related to SDG 3: Good Health and Wellbeing (3, 4). Many of these targets depend on improvements in both in-hospital and out-of-hospital emergency care (5), and will therefore remain out of reach unless there is renewed attention on prehospital settings. In low-and middle-income countries (LMICs), where the majority of deaths can be attributed to disease processes requiring emergency care (6), prehospital medicine represents a markedly neglected field of study (7, 8). Increased focus on improving care in the immediate period following a life-threatening illness or injury before the patient arrives at a healthcare facility, will have a proportionately larger influence on patient outcomes than the advanced care resources further along the chain of survival (9). In a meta-analysis study by Henry and Reingold (10), implementing a formal prehospital care system can reduce the mortality rate from injury by around 25%.

Unfortunately, prehospital emergency care systems (PECS) frequently suffer from resource limitations of both transport and supplies whilst high volumes of patients require urgent care, many of whom are critically ill (8, 11). AI is poised to help tackle these problems because of its unique ability to identify indiscernible patterns and draw conclusions from large amounts of data in a short period of time (12). AI tools that are responsibly developed and incorporated into the PECS have the potential to reform prehospital care by optimising resource allocation and supporting medical staff in time-critical situations (13). Currently, the majority of studies assessing AI use in PECS are conducted in high-income countries (HICs), with most of these studies investigating AI applications in diagnostic and prognostic prediction or optimising cardiac arrest management (14). This study aims to fill a significant research gap by conducting a scoping review that identifies the existing literature on the use of AI within the prehospital emergency medical services (EMS) of LMICs. This focus is in line with the recommendations made by Bedard et al. (7), who have emphasised the necessity for prehospital studies to be undertaken in LMIC settings. Additionally, Razzak et al. (5) advocate for the expanded application of technology in emergency care research in these regions, reinforcing the importance of exploring AI’s potential in enhancing PECS in LMICs and therefore improving a nation’s public health (15). This review will systematically analyse the available evidence, and provide an overview of the extent, range and nature of available evidence on integrating AI technologies into prehospital care systems in LMICs. By reviewing previous AI tools discussed in the published literature, future AI developers, medical researchers and EMS personnel can ensure new models are responsibly adopted, centre around those that will use them and continue to be contextually relevant in the future (16).

## 2 Methods

This scoping review method is described according to the framework outlined by Arksey and O’Malley (17). The study has been reported according the Preferred Reporting Items for Systematic Reviews and Meta-Analyses Extension for Scoping Review (PRISMA-ScR) to ensure a systematic and standardised approach (18).

### 2.1 Identifying the research question

Due to the heterogeneity of study designs and potential topics within the overall theme, a scoping review method was applied (12). The overarching research question that was applied to this study is: what is the current state of artificial intelligence utilisation in prehospital emergency care systems in LMICs within peer-reviewed literature, and what are the implications for future development? This question was meticulously developed and followed PICO guidelines (19). To capture as many potentially relevant articles as possible, we employed a wide approach in research question design, including broader terms when defining the eligibility criteria and searching similar phrases such as “emergency medicine,” “emergency service” and “emergency treatment.” A protocol for this study, combined with a subsequent qualitative study involving the combined thematic analysis of the identified articles from this study and expert interviews, was developed and registered with the Open Science Framework in June 2024 (20).

### 2.2 Identifying relevant studies

Studies were identified through the online databases Medline, Global Health, CINAHL, Embase and Web of Science. Medline, CINAHL, Embase and Web of Science were selected for their extensive coverage of scientific and medical literature. Global Health was chosen as it specialises in international health systems. The search strategy for each electronic database is shown in the Supplementary material. The first literature search was run on 23 May 2024, with another final search carried out on 23 July 2024. Additionally, reference lists of included articles and previously published, relevant literature reviews, such as the scoping review by Chee et al. (14), were hand searched to identify further articles for inclusion.

### 2.3 Study selection

Only primary research studies published in peer-reviewed academic journals were included in the study. Although significant contributions to utilising AI in PECS may exist in grey literature, including in media and at conferences (15, 21), these sources were excluded from the literature search to ensure that any information retrieved for analysis was accurate and of high-quality.

To ensure a comprehensive overview of AI in these settings, whilst simultaneously acknowledging the rapid development of this technology and minimising the risk of including outdated AI applications (12, 22), only studies published from 1 January 2014 until 23 July 2024 were considered. Only articles with full-text available in English were included as the project did not have the ability to translate possibly relevant articles written in other languages into English. Table 1 lays out the inclusion and exclusion criteria for article screening.

**Table 1:** Study inclusion and exclusion criteria.

Criterion	Inclusion criteria	Exclusion criteria
Population	EMS or first responders providing prehospital-or out-of-hospital-accessible emergency care in LMICs, as defined by the World Bank (75).	Paper does not focus on prehospital emergency medicine (e.g., family medicine, urgent care centre etc.). Study does not use data originating from a LMIC.
Intervention	Any computer intervention classified as AI or other similar term by the study authors, including ML and DL.	Author does not specifically identify the model as AI, ML, DL etc. No AI method such as ML or DL is implemented, e.g., only theoretically described.
Comparison	Not required. May involve human experts, other AI tools, statistical models or another comparison group.	None.
Outcome	Any outcome described in the literature.	None.
Publication characteristics	Peer-reviewed journal articles. Article full text available in English. Published 1 January 2014 to 23 July 2024.	Non-peer-reviewed publications, conference proceedings, abstracts, protocols etc. Article full text is not available in English. Articles before 1 January 2014 or after 23 July 2024.

EMS, emergency medical services; LMIC, low-and middle-income country; AI, artificial intelligence; ML, machine learning; DL, deep learning.

All articles identified from the search strategy were extracted into EndNote 21 (Version 21.4) to remove duplicates (23). Articles were then initially single screened by title and abstract. The full text of potentially relevant articles after screening was retrieved and assessed against the eligibility criteria in Table 1. Any uncertainties were resolved by conferring with co-authors. Authors of selected studies were not contacted. Reference lists of selected articles and other relevant literature reviews were then screened by OM, and any potentially applicable articles identified followed the process described above.

### 2.4 Charting the data

Descriptive data from included articles was extracted onto Microsoft Excel 16 (Version 16.87) using a standardised tool designed for charting the data from this study (24). This included article demographics (e.g., author, year of publication etc.), study characteristics (e.g., aim, study design, setting, country, intervention and any comparators, purpose of tool, type of emergency, sample size, outcome measures etc.), study results and the limitations identified by the study authors. Studies were then grouped together based on their overall purpose, with important article demographics, study characteristics and results summarised alongside.

### 2.5 Collating, summarising and reporting the results

The literature review descriptive data is displayed using tables highlighting textual descriptions of the selected studies and appropriate figures. This data was also summarised through a narrative summary.

## 3 Results

**Figure 1:**
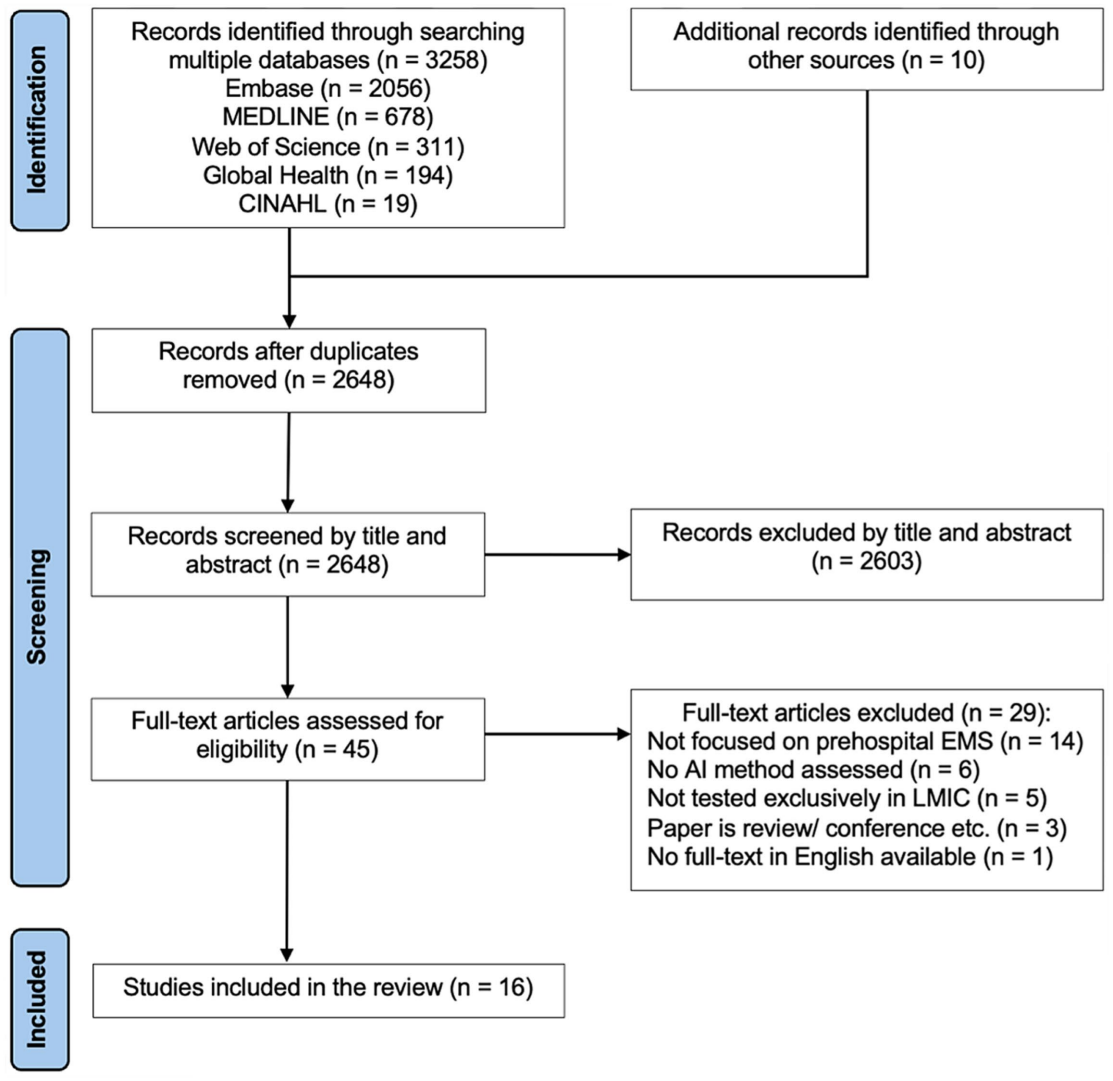
PRISMA flow diagram for literature search. Adapted by the study author based off PRISMA flow diagram from Tricco et al. (18).

Results of the screening process are shown in the PRISMA flow diagram in Figure 1. Database searching identified 3,258 records and a further 10 articles were identified from hand searching the reference lists of included studies and previously published, relevant literature reviews. Once duplicates were removed, 2,648 records underwent screening by title and abstract. After excluding 2,603 articles, 45 articles were retrieved and assessed for full-text eligibility. Out of these articles, the remaining 16 were included in the scoping review.

The characteristics and results of selected studies are included in Table 2. Individual studies have been categorised into three distinct sections: studies related to classification (i.e., categorising records such as calls), dispatch forecasting and/or coordination (studies that target the emergency dispatch or ambulance system), or disease prediction (predicting specific illnesses or injuries) to facilitate comparison between similar interventions. Studies assessing dispatch forecasting/coordination appeared to be the most common studies undertaken (*n* = 8; 50%), followed by disease prediction (*n* = 5; 31%) and classification (*n* = 3; 19%). Most studies have been published from 2021 onwards (*n* = 9; 56%) (25–33), with no studies published before 2016. The year 2022 was the most common year for studies to be published (*n* = 4; 25%) (25, 28, 29, 31).

**Table 2:** Summary table of included studies.

Study	Country	Aim	WHO ECS*	Study design	AI intervention(s)	Comparator	Sample size	Result	Was AI superior?
Classification – Studies that use AI to categorise different records or calls									
Anthony et al. (26)	South Africa	Classifying emergency call transcriptions	Dispatcher	Retrospective cohort	ML: Support vector machine, Random Forest, k-Nearest neighbour, Logistic regression	Dummy (random)	2,326 emergency calls	ML methods had 95% predicted accuracy on unseen data.	N/A – Support vector machine best
Costa et al. (27)	Brazil	Transcribing and classifying unstructured emergency calls	Dispatcher	Retrospective cohort	ML: Automatic speech recognition, Natural language understanding	N/A	93 emergency call transcripts increased to 1,082 using easy data augmentation	Model was accurate with AUCs of emergency call categories ranging from 0.86 to 0.97.	N/A
X. Zhang et al. (28)	China	Classifying prehospital emergency records into groups	Dispatcher	Retrospective cohort	Combined DL model	Various AI tools that are part of combined DL model	37,200 records split into 4 categories	DL model improved the F1 scores by up to 6, 7, 6, and 5% on the four data sets.	Yes – DL model
Dispatch forecasting/ coordination – Studies that focus on the emergency dispatch system and/ or allocate ambulances based on service demand									
Boutilier and Chan (40)	Bangladesh	Predicting emergency transport demand and travel time	Dispatcher and Driver	Retrospective simulation modelling	ML: Random forest, AdaBoost, altered logistic regression, k-Nearest neighbour	N/A	269 ambulance trips, bootstrapped to 4,086	43–64% improvement in prediction accuracy, centralised design using 1/2 the ambulances, small ambulance fleet reduce median response time by 10–18%.	N/A – Random Forest best
Butsingkorn et al. (30)	Thailand	Demand forecasting for ambulance services	Dispatcher	Retrospective cohort	Artificial neural network	AI and non-AI statistical models	25 datasets for each district	Artificial neural network had highest efficiency with the lowest average MAPE.	Yes – Neural network
Huang et al. (37)	China	Forecasting EMS calls using time and weather	Dispatcher	Retrospective cohort	Mixed methods: PNN with non-AI statistical methods	Individual AI and non-AI statistical parts of combined model	365 emergency calls	Significant RMSE decrease and MAPE improvement of the PNN combined model over separate parts.	Yes – PNN best
Ji et al. (38)	China	Redeployment system for ambulances	Driver	Retrospective simulation modelling	Neural network	Random, static redeployment (mathematical optimisation) methods	23,549 EMS request records	Neural network saves around 20% (100 s) of average patient pickup time, improves the ratio of patients picked up within 10 min from 0.786 to 0.838.	Yes – Neural network
Mapuwei et al. (41)	Zimbabwe	Short-term ambulance demand forecasting	Dispatcher	Retrospective cohort	Feed-forward artificial neural network	Various statistical models	96 months of ambulance service data	Neural network MAE superior and RMSE inferior to statistical model. Significant difference between forecast and actual ambulance demand of statistical model but not feed-forward neural network.	Yes – Feed-forward neural network
Rathore et al. (31)	India	Vehicle routing and schedule modelling for EMS	Dispatcher and Driver	Retrospective simulation modelling	ML: Random Forest	Various ML tools	9,766 EMS requests	62 and 14% reduction in the total response time for Random Forest in urban area and rural area, respectively.	N/A – Random Forest best
Torres et al. (32)	Mexico	Classifying travel time estimations returned by a mapping system for ambulances	Driver	Retrospective cohort	ML: Random Forest, Random Forest with hyperparameter, AutoML	Conventional routing applications: Google Maps, Open-Source Routing Machine	2,978 EMS calls for Google Maps; 2,987 EMS calls for OSRM	Google Maps test accuracy was 70% for Random Forest approaches and 72% using AutoML, For Open-Source Routing Machine, performance was 65, 65 and 66% for each ML tool, respectively.	N/A – AutoML best
Yang et al. (33)	China	Forecasting incidence of maritime emergency cases	Dispatcher	Retrospective cohort	ML: Dynamic Bayesian network	Various statistical models	1,312 patients that have undergone a maritime emergency	Statistical model outperformed AI model, with lowest RMSE, MAE, and highest *R^2^*. In most cases, statistical model’s predictions more closely align with the actual number of rescues.	No – Statistical model > Dynamic Bayesian network
Disease prediction – Studies that use AI to predict a specific illness or injury									
Chen et al. (36)	China	Predicting large vessel occlusion strokes with prehospital data	Provider	Retrospective cohort	Artificial neural network	Clinical decision tools	600 patients with acute ischaemic stroke	Artificial neural network AUC, Youden index and accuracy were higher than established prehospital prediction scales.	Yes – Artificial neural network
He et al. (35)	China	Predicting outcome of defibrillation in out of hospital cardiac arrest	Provider	Retrospective cohort	Back-propagation neural network	N/A	199 patients, who received 528 shocks in total	Combining waveform measures improved model performance for subsequent shocks: 10.4, 116.7, 17.3 and 16.4% for AUC, sensitivity, NPV and prediction accuracy, respectively.	N/A
Wang et al. (25)	China	Predicting large vessel occlusion strokes using prehospital-accessible data	Provider	Retrospective cohort	ML: Random forest	Various ML tools: Logistic regression, k-Nearest neighbour, Artificial neural network, gradient boosting machines Clinical decision tools	19, 580 acute ischaemic stroke patients	Random forest and gradient boosting machines AUC of 0.831 (higher than other models). RF had highest specificity (0.827). AUC of RF was higher than other scales.	Yes – Random Forest
Yang et al. (39)	China	Predicting probability of prehospital delay in acute ischaemic stroke patients	Provider and Dispatcher	Prospective cohort	ML: Bayesian network, Support vector machine	Statistical model	450 patients with acute ischaemic stroke	Difference in mean AUC between of best performing ML model and logistic regression model was negligible.	No – Logistic regression = ML
Z. Zhang et al. (29)	China	Predicting stroke mimics in stroke patients in an ambulance	Provider	Retrospective cohort	Artificial neural network: multilayer perceptron	Clinical decision tools	402 patients with suspected or confirmed stroke	AUC of AI model (0.855) was significantly higher than that of clinical decision tools (0.715 and 0.646).	Yes – Artificial neural network

Studies are listed in alphabetical order within each category. EMS, emergency medical services; ML, machine learning; DL, deep learning; PNN, back-propagation neural network-Poisson regression model; AUC, area under receive operator characteristic curve; RMSE, root mean square error; MAE, mean absolute error; R^2^, coefficient of determination; MAPE, mean absolute percentage error; NPV, negative prediction value; N/A, not applicable.

*Refers to which of the Human Resources involved in prehospital care according to the WHO Emergency Care Systems Framework (34) that the AI intervention will assist with.

**Figure 2:**
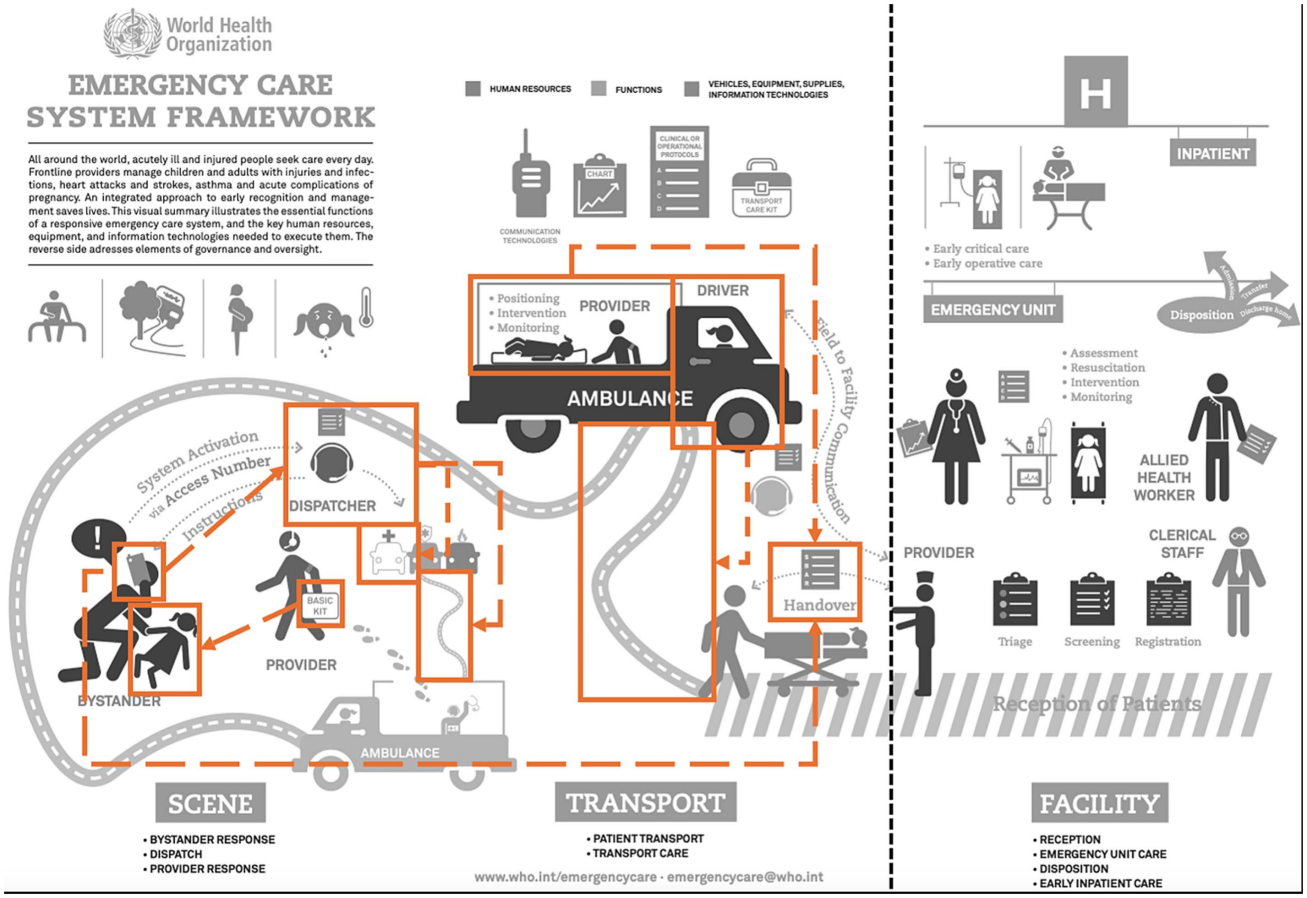
How AI models improve the WHO emergency care system framework. Amended by the author based on original flyer by WHO (34). Permission for the amendments kindly received from emergencycare@who.int on 27-03-2025. Orange boxes and lines reflect the use of AI studies in prehospital EMS. This is an adaptation of an original work “WHO Emergency care system framework. Geneva: World Health Organization (WHO); 2018. Licence: CC BY 4.0.” This adaptation was not created by WHO. WHO is not responsible for the content or accuracy of this adaptation. The original edition shall be the binding and authentic edition.

Figure 2 is an annotated version of the World Health Organization (WHO) Emergency Care System Framework (34), highlighting the areas of PECS that are addressed by the included studies. The dotted black line indicates the point at which emergency care for the patient switches from prehospital (left side of line) to in-hospital care (right side of line). The orange arrows and boxes show how the prehospital AI tools assessed in the selected studies can improve the functions of vehicles, equipment supplies, information technologies or human resources in prehospital emergency care. For example, the neural network that assists in outcome prediction following defibrillation is denoted by the arrow between the basic kit (i.e., a defibrillator) and the unwell patient (i.e., a patient suffering a cardiac arrest) (35). Similarly, the selected studies that investigate classifying unstructured emergency calls are reflected in the link between the bystander’s phone and the dispatcher (26, 27). The selected studies focused the interventions on different human resources staff involved in the PECS, including the dispatcher taking the call from the bystander, the driver bringing the healthcare provider to the patient and then to hospital, and the provider treating the patient.

Most studies were from China (*n* = 9; 56%) (25, 28, 29, 33, 35–39), with no other country having more than one study. Moreover, all the studies assessing disease prediction were conducted in China (*n* = 5; 31%). Although studies were based on data from a range of geographical areas including Latin America, sub-Saharan Africa and across Asia, only studies from middle-income countries (MICs) were identified, with no articles reporting data originating from low-income countries (LICs). For most studies identified in this scoping review, all authors were only affiliated with hospitals or research centres in the country involved in the study, although some studies involved authors affiliated with institutions in other countries (*n* = 4; 25%) (27, 37, 40, 41). Nearly all included studies were retrospective (*n* = 15; 94%), with only one prospective study included in the review (6%) (39). In addition, most studies were cohort studies (*n* = 13; 81%), although three assessed AI tools using simulation modelling (19%) (31, 38, 40). Overall, there was a large variation in sample size, with some studies involving datasets of less than 100 original data points (27), to other studies involving over 37,000 data points (28). However, it is difficult to compare sample sizes due to the heterogenous reporting methods amongst studies. For example, some studies only provided the number of datasets for certain districts or months (30, 41).

Half of the included studies used a deep learning (DL) model as the main intervention (*n* = 8; 50%) (28–30, 35–38, 41), with different types of neural networks applied, including backpropagation (35, 37). Several studies in this review considered the importance of contextually appropriate AI model creation (*n* = 3; 19%) (27, 31, 41). Most studies used some form of comparator to compare the results of the main intervention AI tool (*n* = 12; 75%) (25, 28–33, 36–39, 41), although there was significant variation between the types of comparators, including clinical decision tools, other AI models and mathematical optimisation methods. Out of the studies that involved a comparator, the main AI intervention was superior in all but two studies (*n* = 10; 83%) (33, 39). In the prospective study by Yang et al. (39), there was no significant difference between the machine learning (ML) algorithm and the logistic regression model, however in the retrospective study by Yang et al. (33) the dynamic Bayesian network model was outperformed by the statistical model SARIMA (Seasonal Auto-Regressive Integrated Moving Average).

## 4 Discussion

In this scoping review, 2,648 records published between January 2014 and July 2024 were screened. The study identified 16 primary studies to comprehensively assess the current use of AI in PECS in LMICs. Since 2014, AI has been used in this domain for dispatch forecasting/coordination, disease prediction and information classification. The results of this study represent the first overview of how AI has been used in PECS in LMICs. Research in this area is currently growing, highlighted by the fact that the majority of selected studies have been published in the last 4 years of the over 10-year period considered, and is likely to continue on this trajectory. Overall, models appeared to have a high accuracy, and most models were able to outperform their comparators. One important point to note is that no studies within the literature used datasets gathered in LICs, meaning no studies from LICs have been included in this scoping review. This highlights the gap in research within LMICs between LICs and MICs, limiting the applicability of the study’s findings in these settings. This is similar to the findings in a previous review of prehospital AI use globally (14). This reflects a lack of active research into AI in these countries, possibly due to issues surrounding data accessibility, lack of trained personnel, or funding limitations. Whilst further research beyond academic publications and in grey literature could potentially detect AI use in PECS in LICs, this is beyond the scope of this review. If this research trend is allowed to continue it could have serious implications for the healthcare systems of these LICs, with AI tools disproportionately advancing HICs and MICs and ignoring LICs.

This discussion will outline the findings of this scoping review and identify the possible success factors and potential pitfalls that future developers and researchers may encounter in this field.

### 4.1 The development of AI

The studies selected during the literature review demonstrate the variation in types of AI, including combinations of different models, as well as their broad applicability. As AI tools in PECS continue to be developed in MICs, the current need for successful and sustainably implemented, relevant solutions should guide the principles and future direction of AI research. For the MICs identified in this review, half of the studies discuss AI models for dispatch forecasting and coordination, possibly as limitations to dispatch services such as ambulances are seen as a major obstacle in these settings compared to HICs (8, 42). As AI research continues to develop in LMICs, studies conducted in HICs may serve as reference models for adoption in LMICs and lead to a shift in topics towards those commonly investigated in these settings such as cardiac arrest management and disease prediction. DL models have enabled larger volumes of data to be analysed than previous ML techniques, opening the door for the future rapid development of innovation across a spectrum of datasets including text, audio and video (43, 44). As this technology advances, it seems inevitable that AI will continue to change the field of medicine (45), and based off the study results, prehospital emergency care within that. Some researchers suggest modifying the WHO Emergency Care Systems Framework to recognise the potential of new and advanced IT, including AI, highlighting the global shift towards reliance on this technology and as a way to make the framework more adaptable to future PECS (46). Although the results of this scoping review demonstrate the effective theoretical use of AI, there will be important barriers to overcome before they can be used on real patients. This has been a challenge for many previous AI algorithms (47).

### 4.2 Existing challenges in LMICs

Despite research highlighting the benefits of implementing PECS in LMICs (10, 42, 48), many EMS in these countries currently lack the necessary resources to provide effective care to critically ill and injured patients (7, 8, 49). The results of this study indicate that successfully implemented AI tools can assist LMICs in overcoming the current barriers caused by resource limitations. Ensuring optimal allocation of medical equipment, patient transport and healthcare personnel in PECS using AI tools can assist in promoting the democratisation of quality healthcare in resource-limited settings (50). For example, using AI to optimise ambulance distribution within a city based on the predicted demand, can minimise the ambulance response time and therefore limit the delay before the patient receives prehospital emergency treatment, resulting in reductions in preventable prehospital deaths (51). AI can facilitate these allocation procedures, as well as expand access to medical resources and improve the capabilities of healthcare personnel (52). However, the health problems that currently exist in a given LMIC often result from the unique and complex interplay of many factors that cannot be easily disentangled or categorised (53). In the context of prehospital emergency care, this can include a lack of coordination at the local, national and global levels, differences in research agendas between actors, and limitations in education or training capacity (3, 42, 54). Whilst using AI in a healthcare system may address a single factor contributing to poor health outcomes in a specific setting, it is important to consider the unintended influences this may have on other interacting factors. Although there is some evidence that underdeveloped regions can improve access to healthcare through the use of mobile health and telemedicine services, technology alone will not provide permanent solutions to all the challenges prehospital EMS face in LMICs (55, 56). Improvements to some healthcare systems will require basic economic enhancement before technological investment (56). For example, ML tools to aid in prioritising ambulance dispatch are redundant if they exist in a PECS without emergency vehicles or serve a population that is inaccessible due to a lack of roads. Initial appropriate infrastructure and investment, of both the physical built environment and the IT environment, is compulsory to reap the rewards of AI interventions in PECS in LMICs. This level of preparedness for technological implementation will differ both between and within countries, the context and consequences of which must be fully considered to encourage equitable access for the population. In settings with limited ability to meet computational demands for AI, Yang et al. (33) recommended using a statistical model instead.

### 4.3 Ensuring locally adapted solutions

LMIC is a broad term that describes the majority of the world’s countries and population. No single AI tool can be successfully implemented into the diversity of local environments that make up the countries categorised as low-and middle-income (57). Although AI may promote universal access to health coverage, the democratisation of quality healthcare cannot be realised through the generalised use of unvalidated AI tools (52, 58). Ignoring sociocultural contexts and local adequacy when implementing AI in LMICs has previously resulted in unintended and contextually-inappropriate results (21). One study assessing global access to life-saving skills training across both HICs and LMICs identified cultural beliefs as the most prominent barrier to implementation, highlighting the importance of appropriate sociocultural considerations when applying new PECS solutions to a specific setting (59). Social, political and economic processes play an integral but diverse role in local human health, meaning technological approaches must be context-specific to function correctly (60). Study results originating from a single country cannot be considered blindly generalisable for all LMIC contexts. Most of the studies identified in this scoping review originate from China, which is consistent with other reviews in this field (21, 61). This reflects the country’s dominance in AI research within LMICs and suggests that additional studies conducted in LMICs outside of China are needed. Future funding and research priorities for implementing AI models in PECS must involve local actors and be allocated based on the needs and disease burden of the local population they are designated to help (3, 62). A major advantage of AI is that the technology’s versatility and adaptable techniques allow it to function in a variety of healthcare settings (63). For example, knowledge and AI technology originally developed and implemented in one setting may be appropriately incorporated into another setting, an approach that may open doors to accessing AI tools for LICs (64). Nevertheless, AI tools must be extensively trained and externally validated with unseen, context-specific datasets to minimise contextual bias and maximise applicability (52).

However, to be correctly integrated, AI requires a healthcare system with adequate data availability. Several previous studies have cited challenges with attaining high quality data as a significant issue that must be addressed in LMICs (63, 65–68). AI tools are reliant on data that is accurate, standardised and complete to input into the model in order to ensure decisions will improve patient outcomes (4, 69). Having a sufficient quantity of data in a dataset can improve the model’s diagnostic accuracy and lead to more generalisable results (68). As shown in the results of our study, limited data may have contributed to inaccuracies in some of the AI tools’ predictive ability, highlighting the need for large but local datasets to train AI models for precise prediction (33, 39). This data is often missing in resource-poor settings, meaning acquiring complete datasets is a significant barrier to the successful development and implementation of AI tools in many LMICs and can result in missing data bias (51, 63). Furthermore, some of the studies identified in this scoping review are inadequately reported, and the heterogenous reporting methods for data within the studies prevents direct comparison between studies. Future studies should ensure data is presented in a standardised format using reporting guidelines such as the CONSORT-AI (Consolidated Standards of Reporting Trials – Artificial Intelligence) guidelines (70). This can alleviate data collection bias and make sure models are trained on high quality data, both seen as key actions that limit data bias by some of the studies in this scoping review (25, 27, 31, 33).

### 4.4 Future directions

The results of this study suggest that AI tools may play a key role in future digital public health solutions by seeking to reduce individual morbidity and mortality in out-of-hospital settings as a means of improving population health. However, as shown by this scoping review, the current research field is dominated by retrospective, small-sample studies. Further prospective and randomised studies using larger, accurate datasets in the prehospital settings of LMICs are needed to ensure the samples used for research more accurately reflect the real-life population. Although prospective studies are important in all healthcare research to reduce the risk of bias and confounding, they are especially important in studies assessing AI models. AI algorithms often overfit retrospective datasets and therefore over-estimate model accuracy, leading to underperformance when tested against real-world data (21, 63). This phenomenon may have been reflected in our study results, with the only prospective trial included noting no significant difference between the performances of the AI model and the statistical model (39).

As more and more studies are published every year using AI solutions in the EMS, additional investments in research should prioritise LICs that are currently being excluded within the published literature to help slow and eventually reverse the widening gap in global health inequity. This investment must include expanding access to suitable data collection methods to promote larger, prospective studies in this field such as facilitating the installation of electronic health records and educating prehospital staff in data collection procedures. One option to promote the development of AI in LICs, is using AI tools previously conceived in other settings and leveraging off the already completed intermediary steps, with proper adjustment for the local context. This reduces the burden of new technology development that can be expensive and resource intensive, allowing wider access to this technology, including in countries that currently lack this initial development ability (64). AI systems can also leapfrog off previously integrated mobile health initiatives by utilising the technology already implemented to speed up setting up these tools. These recommendations can be successfully implemented through increased international collaboration between LICs, and MICs and HICs. These partnerships can be co-ordinated through the WHO and its already established networks, such as the Acute Care Action Network. This global alliance can use AI as a means of achieving some of their key operation priorities, including strengthening acute care services and improving clinical quality (71). HICs and technology companies involved in AI and emergency care development can provide technology and knowledge transfer, with other international organisations such as the World Bank offering cooperation grants to ease the financial burden (64). Any current partnerships, such as those identified in this scoping review, should be encouraged and may act as a springboard for future AI research in these countries.

Future studies should also only incorporate data gathered from the local population. Whilst, in some cases, this may contribute to reductions in the quantity of available data to design and test models, this approach can help promote relevant and contextual models that are adapted to the system and population into which they will be implemented. As AI design continues to advance with new, more powerful layouts such as large language models, and the technology becomes more accessible through the increased availability of lower cost models, AI may support PECS in the future in previously unforeseen ways. However, irrespective of the complexity of the technology used in future models or its function, qualitative research investigating the use of AI in PECS in LMICs from a variety of relevant perspectives including researchers, clinicians and patients is essential in understanding key implications for successful future development. These additional studies can cement the principle of human-centred AI design within future AI models.

### 4.5 Limitations

This study has several limitations. Firstly, our study only assessed articles published in English, potentially excluding otherwise relevant articles. This is a particularly important limitation to consider for studies originating from China, one of the global leaders in AI research (21) and the country with the most studies in this scoping review. Secondly, only studies in which the author classified the model used as a form of AI were included. This may result in several relevant articles being excluded during the screening process. Furthermore, studies that did not identify as being from a LMIC in their title or abstract may have been missed by the search strategy. Although hand searching reference lists identified some studies that fall under this category, it is possible that others were missed. In addition, as well as prospective and retrospective studies, this study included simulation models in data analysis which may limit the external validity of the findings (72). Simulations were included in this study because AI in PECS remains an emerging field, therefore these findings can still be useful to guide future studies. Finally, as this is a scoping review, there was no methodological critical appraisal of the individual studies, possibly limiting the applicability of recommendations for policy (73).

## 5 Conclusion

AI is seen by some to be a key missing piece in the drive towards universal health coverage and the realisation of SDG 3: Good Health and Wellbeing (4, 54, 58). Improvements across all areas of medicine, but particularly in prehospital emergency care will be an essential step to achieve this ambitious target (74). Currently, AI models generally outperform other tools in simulation and cohort studies in the field of PECS within MICs, however there are currently no studies using an AI tool in PECS in LIC settings. To ensure these models benefit the patients, staff and healthcare systems they are used in, the data that is collected to train and test the models must be high-quality and context specific, and models should be designed with appropriate consideration of all end-users. Furthermore, AI researchers should consider the environment into which AI tools are being implemented ensure models are contextually appropriate. Future research should be supported by improved international collaboration and should focus on large, prospective studies from a diverse range of LMICs, with additional support made available for LICs. This can ensure AI becomes part of the solution to the challenges faced by prehospital EMS in LMICs, and by extension, the drive for global health equity.

## Data Availability

The original contributions presented in the study are included in the article/Supplementary material, further inquiries can be directed to the corresponding authors.

## References

[1] Secinaro S Calandra D Secinaro A Muthurangu V Biancone P. The role of artificial intelligence in healthcare: a structured literature review. BMC Med Inform Decis Mak. (2021) 21:125. doi: 10.1186/s12911-021-01488-9, PMID: 33836752 PMC8035061

[2] Vinuesa R Azizpour H Leite I Balaam M Dignum V Domisch S . The role of artificial intelligence in achieving the sustainable development Goals. Nat Commun. (2020) 11:233. doi: 10.1038/s41467-019-14108-y, PMID: 31932590 PMC6957485

[3] Schwalbe N Wahl B. Artificial intelligence and the future of global health. Lancet. (2020) 395:1579–86. doi: 10.1016/S0140-6736(20)30226-9, PMID: 32416782 PMC7255280

[4] Singh JA. Artificial intelligence and global health: opportunities and challenges. Emerg Top Life Sci. (2019) 3:741–6. doi: 10.1042/ETLS20190106 32915223

[5] Razzak J Beecroft B Brown J Hargarten S Anand N. Emergency care research as a global health priority: key scientific opportunities and challenges. BMJ Glob Health. (2019) 4:e001486. doi: 10.1136/bmjgh-2019-001486, PMID: 31406602 PMC6666807

[6] Razzak J Usmani MF Bhutta ZA. Global, regional and national burden of emergency medical diseases using specific emergency disease indicators: analysis of the 2015 global burden of disease study. BMJ Glob Health. (2019) 4:e000733. doi: 10.1136/bmjgh-2018-000733, PMID: 30997158 PMC6441258

[7] Bedard AF Mata LV Dymond C Moreira F Dixon J Schauer SG . A scoping review of worldwide studies evaluating the effects of prehospital time on trauma outcomes. Int J Emerg Med. (2020) 13:64. doi: 10.1186/s12245-020-00324-7, PMID: 33297951 PMC7724615

[8] Bhattarai HK Bhusal S Barone-Adesi F Hubloue I. Prehospital emergency care in low-and middle-income countries: a systematic review. Prehosp Disaster Med. (2023) 38:495–512. doi: 10.1017/S1049023X23006088, PMID: 37492946 PMC10445116

[9] Deakin CD. The chain of survival: not all links are equal. Resuscitation. (2018) 126:80–2. doi: 10.1016/j.resuscitation.2018.02.012, PMID: 29471008

[10] Henry JAM Reingold ALM. Prehospital trauma systems reduce mortality in developing countries: a systematic review and meta-analysis. J Trauma Acute Care Surg. (2012) 73:261–8. doi: 10.1097/TA.0b013e31824bde1e, PMID: 22743393

[11] Piliuk K Tomforde S. Artificial intelligence in emergency medicine. A systematic literature review. Int J Med Inform. (2023) 180:105274. doi: 10.1016/j.ijmedinf.2023.105274, PMID: 37944275

[12] Masoumian Hosseini M Masoumian Hosseini ST Qayumi K Ahmady S Koohestani HR. The aspects of running artificial intelligence in emergency care; a scoping review. Arch Acad Emerg Med. (2023) 11:e38. doi: 10.22037/aaem.v11i1.1974, PMID: 37215232 PMC10197918

[13] Cimino J Braun C. Clinical research in prehospital care: current and future challenges. Clin Pract. (2023) 13:1266–85. doi: 10.3390/clinpract13050114, PMID: 37887090 PMC10605888

[14] Chee ML Chee ML Huang H Mazzochi K Taylor K Wang H . Artificial intelligence and machine learning in prehospital emergency care: a scoping review. iScience. (2023) 26:107407. doi: 10.1016/j.isci.2023.107407, PMID: 37609632 PMC10440716

[15] Wahl B Cossy-Gantner A Germann S Schwalbe NR. Artificial intelligence (AI) and global health: how can AI contribute to health in resource-poor settings? BMJ Glob Health. (2018) 3:e000798. doi: 10.1136/bmjgh-2018-000798, PMID: 30233828 PMC6135465

[16] Hadley TD Pettit RW Malik T Khoei AA Salihu HM. Artificial intelligence in global health-a framework and strategy for adoption and sustainability. Int J MCH AIDS. (2020) 9:121–7. doi: 10.21106/ijma.296, PMID: 32123635 PMC7031870

[17] Arksey H O'Malley L. Scoping studies: towards a methodological framework. Int J Soc Res Methodol. (2005) 8:19–32. doi: 10.1080/1364557032000119616

[18] Tricco AC Lillie E Zarin W O'Brien KK Colquhoun H Levac D . PRISMA extension for scoping reviews (PRISMA-ScR): checklist and explanation. Ann Intern Med. (2018) 169:467–73. doi: 10.7326/M18-0850, PMID: 30178033

[19] Richardson WS Wilson MC Nishikawa J Hayward RS. The well-built clinical question: a key to evidence-based decisions. ACP J Club. (1995) 123:A12–3. doi: 10.7326/ACPJC-1995-123-3-A12, PMID: 7582737

[20] Mallon O Pilot E Lippert F. Utilising artificial intelligence in the prehospital emergency medical services of LMICs: a scoping review. OSF (2024).

[21] Ciecierski-Holmes T Singh R Axt M Brenner S Barteit S. Artificial intelligence for strengthening healthcare systems in low-and middle-income countries: a systematic scoping review. NPJ Digit Med. (2022) 5:162. doi: 10.1038/s41746-022-00700-y, PMID: 36307479 PMC9614192

[22] Ali O Abdelbaki W Shrestha A Elbasi E Alryalat MAA Dwivedi YK. A systematic literature review of artificial intelligence in the healthcare sector: benefits, challenges, methodologies, and functionalities. J Innov Knowl. (2023) 8:100333. doi: 10.1016/j.jik.2023.100333

[23] Clarivate EndNote 21.21.4. Clarivate (2024)

[24] Microsoft Corporation. Microsoft Excel for Mac. 16.87. (2024).

[25] Wang J Zhang J Gong X Zhang W Zhou Y Lou M. Prediction of large vessel occlusion for ischaemic stroke by using the machine learning model random forests. Stroke Vasc Neurol. (2022) 7:94–100. doi: 10.1136/svn-2021-001096, PMID: 34702747 PMC9067264

[26] Anthony T Mishra AK Stassen W Son J. The feasibility of using machine learning to classify calls to south African emergency dispatch centres according to prehospital diagnosis, by utilising caller descriptions of the incident. Healthcare. (2021) 9:1107. doi: 10.3390/healthcare9091107, PMID: 34574881 PMC8472370

[27] Costa DB Pinna FCA Joiner AP Rice B Souza JVP Gabella JL . AI-based approach for transcribing and classifying unstructured emergency call data: a methodological proposal. PLoS Digit Health. (2023) 2:e0000406. doi: 10.1371/journal.pdig.0000406, PMID: 38055710 PMC10699611

[28] Zhang X Zhang H Sheng L Tian F. DL-PER: deep learning model for Chinese prehospital emergency record classification. IEEE Access. (2022) 10:64638–49. doi: 10.1109/ACCESS.2022.3179685

[29] Zhang Z Zhou D Zhang J Xu Y Lin G Jin B . Multilayer perceptron-based prediction of stroke mimics in prehospital triage. Sci Rep. (2022) 12:17994. doi: 10.1038/s41598-022-22919-1, PMID: 36289277 PMC9606292

[30] Butsingkorn T Apichottanakul A Arunyanart S. Predicting demand for emergency ambulance services: a comparative approach. J Appl Sci Eng. (2024) 27:3313–8. doi: 10.6180/jase.202410_27(10).0011

[31] Rathore N Jain PK Parida M. A sustainable model for emergency medical Services in Developing Countries: a novel approach using partial outsourcing and machine learning. Risk Manag Healthc Policy. (2022) 15:193–218. doi: 10.2147/RMHP.S338186, PMID: 35173497 PMC8841749

[32] Torres N Trujillo L Maldonado Y Vera C. Correction of the travel time estimation for ambulances of the red cross Tijuana using machine learning. Comput Biol Med. (2021) 137:104798. doi: 10.1016/j.compbiomed.2021.104798, PMID: 34482200

[33] Yang P Cheng P Zhang N Luo D Xu B Zhang H. Statistical machine learning models for prediction of China's maritime emergency patients in dynamic: ARIMA model, SARIMA model, and dynamic Bayesian network model. Front Public Health. (2024) 12:1401161. doi: 10.3389/fpubh.2024.1401161, PMID: 39022407 PMC11252837

[34] WHO. WHO emergency care system framework. Geneva: WHO (2018).

[35] He M Lu Y Zhang L Zhang H Gong Y Li Y. Combining amplitude Spectrum area with previous shock information using neural networks improves prediction performance of defibrillation outcome for subsequent shocks in out-of-hospital cardiac arrest patients. PLoS One. (2016) 11:e0149115. doi: 10.1371/journal.pone.0149115, PMID: 26863222 PMC4749245

[36] Chen Z Zhang R Xu F Gong X Shi F Zhang M . Novel prehospital prediction model of large vessel occlusion using artificial neural network. Front Aging Neurosci. (2018) 10:181. doi: 10.3389/fnagi.2018.00181, PMID: 29997494 PMC6028566

[37] Huang H Jiang M Ding Z Zhou M. Forecasting emergency calls with a Poisson neural network-based assemble model. IEEE Access. (2019) 7:18061–9. doi: 10.1109/ACCESS.2019.2896887

[38] Ji S Zheng Y Wang Z Li T. A deep reinforcement learning-enabled dynamic redeployment system for Mobile ambulances. Proc ACM Interact Mob Wearable Ubiquitous Technol. (2019) 3:1–20. doi: 10.1145/3314402, PMID: 39076787

[39] Yang L Liu Q Zhao Q Zhu X Wang L. Machine learning is a valid method for predicting prehospital delay after acute ischemic stroke. Brain Behav. (2020) 10:e01794. doi: 10.1002/brb3.1794, PMID: 32812396 PMC7559608

[40] Boutilier JJ Chan TCY. Ambulance emergency response optimization in developing countries. Oper Res. (2020) 68:1315–34. doi: 10.1287/opre.2019.1969, PMID: 19642375

[41] Mapuwei TW Bodhlyera O Mwambi H. Univariate time series analysis of short-term forecasting horizons using artificial neural networks: the case of public ambulance emergency preparedness. J Appl Math. (2020) 2020:1–11. doi: 10.1155/2020/2408698

[42] Kironji AG Hodkinson P de Ramirez SS Anest T Wallis L Razzak J . Identifying barriers for out of hospital emergency care in low and low-middle income countries: a systematic review. BMC Health Serv Res. (2018) 18:291. doi: 10.1186/s12913-018-3091-0, PMID: 29673360 PMC5907770

[43] Mendo IR Marques G de la Torre Díez I López-Coronado M Martín-Rodríguez F. Machine learning in medical emergencies: a systematic review and analysis. J Med Syst. (2021) 45:88. doi: 10.1007/s10916-021-01762-3, PMID: 34410512 PMC8374032

[44] Wason R. Deep learning: evolution and expansion. Cogn Syst Res. (2018) 52:701–8. doi: 10.1016/j.cogsys.2018.08.023

[45] Hamedani Z Moradi M Kalroozi F Manafi Anari A Jalalifar E Ansari A . Evaluation of acceptance, attitude, and knowledge towards artificial intelligence and its application from the point of view of physicians and nurses: a provincial survey study in Iran: a cross-sectional descriptive-analytical study. Health Sci Rep. (2023) 6:e 1543. doi: 10.1002/hsr2.1543, PMID: 37674620 PMC10477406

[46] Böbel S Verhoeven J Scholz M Penders B Frisina Doetter L Collatz Christensen H . Strengthening the WHO emergency care systems framework: insights from an integrated, patient-centered approach in the Copenhagen emergency medical services system—a qualitative system analysis. BMC Health Serv Res. (2025) 25:401. doi: 10.1186/s12913-025-12465-7, PMID: 40102833 PMC11916934

[47] Mueller B Kinoshita T Peebles A Graber MA Lee S. Artificial intelligence and machine learning in emergency medicine: a narrative review. Acute Med Surg. (2022) 9:e740. doi: 10.1002/ams2.740, PMID: 35251669 PMC8887797

[48] Razzak JA Kellermann AL. Emergency medical care in developing countries: is it worthwhile? Bull World Health Organ. (2002) 80:900–5. Available at: https://pmc.ncbi.nlm.nih.gov/articles/PMC2567674/12481213 PMC2567674

[49] Schell CO Gerdin Wärnberg M Hvarfner A Höög A Baker U Castegren M . The global need for essential emergency and critical care. Crit Care. (2018) 22:284. doi: 10.1186/s13054-018-2219-2, PMID: 30373648 PMC6206626

[50] Alami H Rivard L Lehoux P Hoffman SJ Cadeddu SBM Savoldelli M . Artificial intelligence in health care: laying the foundation for responsible, sustainable, and inclusive innovation in low-and middle-income countries. Glob Health. (2020) 16:52. doi: 10.1186/s12992-020-00584-1, PMID: 32580741 PMC7315549

[51] Chenais G Lagarde E Gil-Jardiné C. Artificial intelligence in emergency medicine: viewpoint of current applications and foreseeable opportunities and challenges. J Med Internet Res. (2023) 25:e40031. doi: 10.2196/40031, PMID: 36972306 PMC10245226

[52] Weissglass DE. Contextual bias, the democratization of healthcare, and medical artificial intelligence in low-and middle-income countries. Bioethics. (2022) 36:201–9. doi: 10.1111/bioe.12927, PMID: 34460977

[53] Engel N Hoyweghen IV Krumeich A. Making global health care innovation work. New York: Palgrave Macmillan (2014).

[54] Guo J Li B. The application of medical artificial intelligence Technology in Rural Areas of developing countries. Health Equity. (2018) 2:174–81. doi: 10.1089/heq.2018.0037, PMID: 30283865 PMC6110188

[55] Liu X Chen W Qiu Y Li X Liu F Jiang Z . Improving access to cardiovascular care for 1.4 billion people in China using telehealth. NPJ Digit Med. (2024) 7:376. doi: 10.1038/s41746-024-01381-5, PMID: 39715810 PMC11666715

[56] Delaney PG Moussally J Wachira BW. Future directions for emergency medical services development in low-and middle-income countries. Surgery. (2024) 176:220–2. doi: 10.1016/j.surg.2024.02.030, PMID: 38599983

[57] Jouffroy R Djossou F Neviere R Jaber S Vivien B Heming N . The chain of survival and rehabilitation for sepsis: concepts and proposals for healthcare trajectory optimization. Ann Intensive Care. (2024) 14:58. doi: 10.1186/s13613-024-01282-6, PMID: 38625453 PMC11019190

[58] Parsa AD Hakkim S Vinnakota D Mahmud I Bulsari S Dehghani L . Artificial intelligence for global healthcare In: Moy Chatterjee J Saxena SK, editors. Artificial Intelligence in Medical Virology. Singapore: Springer Nature Singapore (2023). 1–21.

[59] Stassen W Chern YL Blewer AL Kong SYJ Lippert F Ong MEH . Barriers and facilitators to global access to life-saving skills training: an international cross-sectional survey. BMJ Open. (2025) 15:e090562. doi: 10.1136/bmjopen-2024-090562, PMID: 39880417 PMC11781109

[60] Leach M Scoones I. Health dynamics, innovation and the slow race to make technology work for the poor. London: Pro-Book Publishing; (2008). 124–127.

[61] Meyer LM Stead S Salge TO Antons D. Artificial intelligence in acute care: a systematic review, conceptual synthesis, and research agenda. Technol Forecast Soc Change. (2024) 206:123568. doi: 10.1016/j.techfore.2024.123568

[62] Lecky FE Reynolds T Otesile O Hollis S Turner J Fuller G . Harnessing inter-disciplinary collaboration to improve emergency care in low-and middle-income countries (LMICs): results of research prioritisation setting exercise. BMC Emerg Med. (2020) 20:68. doi: 10.1186/s12873-020-00362-7, PMID: 32867675 PMC7457362

[63] Christie SA Hubbard AE Callcut RA Hameed M Dissak-Delon FN Mekolo D . Machine learning without borders? An adaptable tool to optimize mortality prediction in diverse clinical settings. J Trauma Acute Care Surg. (2018) 85:921–7. doi: 10.1097/TA.0000000000002044, PMID: 30059457 PMC6225991

[64] Khan MS Umer H Faruqe F. Artificial intelligence for low income countries. Humanit Soc Sci Commun. (2024) 11:1422. doi: 10.1057/s41599-024-03947-w

[65] Zuhair V Babar A Ali R Oduoye MO Noor Z Chris K . Exploring the impact of artificial intelligence on Global Health and enhancing healthcare in developing nations. J Prim Care Community Health. (2024) 15:21501319241245847. doi: 10.1177/21501319241245847, PMID: 38605668 PMC11010755

[66] Vishwakarma LP Singh RK Mishra R Kumari A. Application of artificial intelligence for resilient and sustainable healthcare system: systematic literature review and future research directions. Int J Prod Res. (2023) 63:1–23. doi: 10.1080/00207543.2023.2188101

[67] Kelly CJ Karthikesalingam A Suleyman M Corrado G King D. Key challenges for delivering clinical impact with artificial intelligence. BMC Med. (2019) 17:195. doi: 10.1186/s12916-019-1426-2, PMID: 31665002 PMC6821018

[68] Jayakumar S Sounderajah V Normahani P Harling L Markar SR Ashrafian H . Quality assessment standards in artificial intelligence diagnostic accuracy systematic reviews: a meta-research study. NPJ Digit Med. (2022) 5:11. doi: 10.1038/s41746-021-00544-y, PMID: 35087178 PMC8795185

[69] O'Neil S Taylor S Sivasankaran A. Data equity to advance health and health equity in low-and middle-income countries: a scoping review. Digit Health. (2021) 7:20552076211061922. doi: 10.1177/20552076211061922, PMID: 34992789 PMC8725220

[70] Liu X Cruz Rivera S Moher D Calvert MJ Denniston AK Chan A-W . Reporting guidelines for clinical trial reports for interventions involving artificial intelligence: the CONSORT-AI extension. Nat Med. (2020) 26:1364–74. doi: 10.1038/s41591-020-1034-x, PMID: 32908283 PMC7598943

[71] WHO. (2025) Acute care action network (ACAN). Available online at: https://www.who.int/groups/acute-care-action-network (Accessed May 07, 2025).

[72] Lamé G Simmons RK. From behavioural simulation to computer models: how simulation can be used to improve healthcare management and policy. BMJ Simul Technol Enhanc Learn. (2020) 6:95–102. doi: 10.1136/bmjstel-2018-000377, PMID: 35516085 PMC8936879

[73] Woo BFY Tam WWS Williams MY Ow Yong JQY Cheong ZY Ong YC . Characteristics, methodological, and reporting quality of scoping reviews published in nursing journals: a systematic review. J Nurs Scholarsh. (2023) 55:874–85. doi: 10.1111/jnu.12861, PMID: 36494752

[74] Schnaubelt S Garg R Atiq H Baig N Bernardino M Bigham B . Cardiopulmonary resuscitation in low-resource settings: a statement by the international liaison committee on resuscitation, supported by the AFEM, EUSEM, IFEM, and IFRC. Lancet Glob Health. (2023) 11:e1444–53. doi: 10.1016/S2214-109X(23)00302-9, PMID: 37591590

[75] The World Bank. (2024) World Bank country and lending groups 2024. Available online at: https://datahelpdesk.worldbank.org/knowledgebase/articles/906519-world-bank-country-and-lending-groups (Accessed March 25, 2024).

